# Acute Colitis Caused by *Helicobacter trogontum* in Immunocompetent Patient

**DOI:** 10.3201/eid2202.150287

**Published:** 2016-02

**Authors:** Fabien Dutasta, Elia Samaha, Nathalie Carayol, Jean-Marc Masse, Camille Bourillon, Clémence Richaud, Arthur Neuschwander, Hidayeth Rostane, Marie Lyse Parolini, Patrick Bruneval, Christophe Cellier, Isabelle Podglajen

**Affiliations:** Hôpital d’Instruction des Armées Legouest, Metz, France (F. Dutasta);; Hôpital Européen Georges Pompidou, Paris, France (E. Samaha, C. Bourillon, A. Neuschwander, H. Rostane, M.L. Parolini, P. Bruneval, C. Cellier, I. Podglajen);; Collège de France, Paris (N. Carayol, I. Podglajen);; Electron Microscopy Platform, Cochin Institute, Paris (J.M. Masse);; Hôpital Beaujon, Paris (C. Richaud);; Université Paris Descartes, Paris (P. Bruneval, C. Cellier, I. Podglajen);; Institut National de la Santé et de la Recherche Médicale, Paris (P. Bruneval, C. Cellier, I. Podglajen)

**Keywords:** colitis, bacteremia, non–*pylori Helicobacter*, Caco-2 cell cycle arrest, inflammatory bowel disease, bacteria, Helicobacter trogontum

**To the Editor:** In industrialized countries, diarrhea and vomiting associated with acute gastrointestinal illness is estimated to occur at a rate of ≈1 episode per person per year; ≈0.3% of patients are hospitalized because of severe symptoms associated with colitis or fever. The most commonly identified infectious agents of non-nosocomial diarrhea are calicivirus and *Salmonella*, *Campylobacter*, *Giardia*, and *Cryptosporidium *spp. However, for numerous cases, the causative agent is not identified. *Helicobacter *species other than *H. pylori*, but not *H. trogontum*, have emerged as causes of gastrointestinal and systemic disease, mainly in immunocompromised patients (*1*). We report a case of community-acquired colitis with bacteremia caused by *H. trogontum* in an immunocompetent patient and emphasize the diagnostic difficulties.

The patient was a 31-year-old woman with a history of recurrent epigastralgia, vomiting, diarrhea, and weight loss of 10 kg over an 8-year period. In April 2014, she was admitted to the Hôpital Européen Georges Pompidou emergency ward (Paris, France) because of abdominal pain, nonbloody diarrhea, fever, and chills, which had persisted for 3 days. Examination revealed a mildly tender abdomen without hepatosplenomegaly, signs of slight dehydration, and tachycardia. Leukocyte count was 13.2 × 10^9^ cells/L (neutrophils, 11.4× 10^9^), and C-reactive protein level was 191 mg/L. Abdominal computed tomography images showed nonspecific right and transverse colitis (Figure, panel A).

**Figure:**
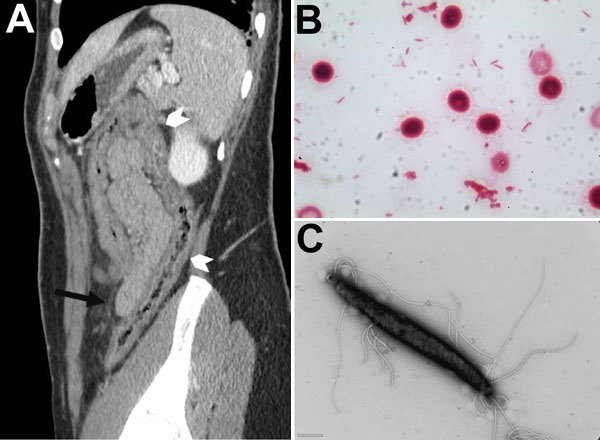
Computed tomographic image of patient with *Helicobacter trogontum *infection and micrographs of *H. trogontum.* A) Paramedian sagittal section of an abdominopelvic scan after injection of contrast medium in the portal phase, showing thickening of the transverse and right colon (white arrowheads) with tubular appearance and discrete thickening of the fat stranding (black arrow). B) Gram-stained blood culture smear. Original magnification ×1,000. C) Transmission electron micrograph of negatively stained *H. trogontum* showing bipolar flagella**. **Scale bar indicates 0.5 μm.

One day after admission, the patient was discharged with empirically prescribed ciprofloxacin and metronidazole for 7 days. After 4 days, aerobic blood culture was positive for motile, fusiform, gram-negative bacilli, suggestive of strictly aerobic bacteria that could not be identified directly (Figure, panels B, C). After 7 days of incubation under a microaerophilic atmosphere only, a blood subculture isolate was obtained; 23S and 16S rDNA sequencing (Technical Appendix) identified this isolate as *H. trogontum*. Of note, use of matrix-assisted laser desorption/ionization time-of-flight mass spectrometry (Bruker Daltonik GmbH, Bremen, Germany) did not enable identification of the bacterium.

No common pathogens were detected in fecal samples. Upper and lower gastrointestinal endoscopic examinations conducted 1 month after discharge revealed no notable abnormalities. No immunocompromised condition was found. At most recent follow-up examination, the patient was free of symptoms.

The genus *Helicobacter* currently comprises 48 formally named species belonging to the gastric or enterohepatic group according to their ecologic niche. *H. trogontum* (enterohepatic group) has been isolated from apparently health animals (rat and piglet intestinal mucosa and swine feces), but its characteristics are typical of pathogenic bacteria ([*2**,**3*]*,* Technical Appendix). The apparent in vitro susceptibility of the isolate to metronidazole and the favorable patient outcome reported here are in agreement with the finding that metronidazole is an effective treatment for *H. trogontum* infection in rats ([*4*]; Technical Appendix), but there are no antimicrobial drug susceptibility data for *H. trogontum *isolated from animals. We assume that the immunocompetent patient reported here had chronic colitis caused by *H. trogontum*, followed by an episode of acute colitis with bacteremia after several years of intermittent symptoms.

The rarity of reported *H. trogontum* infections might be linked to the difficulty associated with culturing and identifying the bacterium or to a low level of exposure to this pathogen. The mode of transmission, probably from animals to humans, remains unclear. Methods for isolation and rapid identification of *H. trogontum*, including the updating of matrix-assisted laser desorption/ionization time-of-flight mass spectrometry databases, are needed for further elucidation of its pathogenic properties and the mode of contamination.

**Technical Appendix.** Supplemental methods for identifying *Helicobacter trogontum* and transmission electron micrograph of *H. trogontum* and Caco-2 cell challenged with *H. trogontum*.
