# Supplementary material for: Acute Colitis Caused by Helicobacter trogontum in Immunocompetent Patient
Source: Emerg Infect Dis. 2016 Feb;22(2):335–6. doi: 10.3201/eid2202.150287 (PMC4734537; doi:10.3201/eid2202.150287)
Supplement: Supplementary file 1 — Technical Appendix. Supplemental methods for identifying Helicobacter trogontum and transmission electron micrograph of H. trogontum and Caco-2 cell challenged with H. trogontum. [file 15-0287-Techapp-s1.pdf]

Article DOI: http://dx.doi.org/10.3201/eid2202.150287

Acute Colitis Caused by *Helicobacter trogontum* in Immunocompetent Patient

Technical Appendix

Helicobacter trogontum Culture, Identification, and Antibiotic Apparent Susceptibility

A set of aerobic and anaerobic blood culture bottles was incubated at 35°C using the BactT/ALERT 3D DUAL-T system (bioMérieux, Marcy l'Étoile, France). Aerobic blood culture was positive after 4 days with motile, fusiform and slightly curved gram-negative bacilli (Figure B). Subculture of the aerobic sample was positive after 7 days of incubation on chocolate or sheep blood (5% v/v) agar only under microaerophilic conditions; for subsequent cultures, incubation for 4 days was sufficient. In the case of blood infection with *Helicobacter cinaedi*, Araoka et al. showed that the time required for the blood culture to become positive ranged from 2 to 12 days (*1*). If non–*H. pylori Helicobacter* species (NHPH) are searched for, the clinician and microbiologist must be aware that the duration of monitored blood cultures should be extended. Currently, there are no selective media or molecular methods for the rapid detection and identification of *H. trogontum*, as well as other NHPH, in clinical samples, including feces and digestive biopsies.

Growth occurred at 35°C and 42°C and appeared in the form of grayish colonies spreading across the agar surface. The isolate had catalase, oxidase and urease activities, as shown by Mendes et al. (ref. 2 cited in the article). Cultures older than ≈9 days consisted of coccoid forms that were nonviable. Matrix-assisted laser desorption ionization time-of-flight mass spectrometry (MALDI-TOF MS; Bruker Daltonics, Billerica, MA, USA) did not enable identification of the bacteria directly from cultured or subcultured blood samples and did not reveal any relationship with the genus *Helicobacter* although *H. pylori*, *H. canadensis*, *H. canis*, *H. cholecystus*, *H. cinaedi*, *H. fenneliae* and *H. mustelae* were represented in the Bruker MS database. Molecular identification of *H. trogontum* was made, after DNA purification using InstaGene Matrix (Bio-Rad, Hercules, CA, USA), using 16S and 23S rDNA-specific primers (*2*,*3*). Sequences were compared to those in the GeneBank database using BIBI[PQP] and SepsiTest blast programs (https://umr5558-bibiserv.univ-lyon1.fr/lebibi/lebibi.cgi, http://www.sepsitest-blast.de/en/index.html). The 16S and 23S rDNA exhibited 98.4% identity with that of *H. trogontum*

strain ATCC700114 isolated from rat (GenBank accession number U65103). *H. trogontum* was originally classified as *Flexispira* Taxon 1, 4, 5, 6 and also comprised in the heterogeneous entity called *Helicobacter rappini* (*4*).

H. trogontum presents multiple bipolar active flagella (7 on the present isolate) and periplasmic fibers (Figure C, Supplemental Figure A, B). This finding explains its ability to move in mucus and to interact with enterocytes.

After 4 days of incubation on blood agar, the isolate analyzed here showed inhibition zone diameters of >50 mm for rifampin and tetracycline and 37 mm for metronidazole. The isolate also had MICs for amoxicillin, ciprofloxacin, and clarithromycin of respectively 2, 32 and 0.38 g/L.

Caco-2/TC7 Cell Experiments

H. trogontum induces subclinical inflammatory bowel disease (IBD) in germ-free mice and acute-to-chronic typhlocolitis with weight loss and diarrhea in B6-IL-10$^{-/-}$ mice, in which it causes loss of microvilli and risk of sepsis. This risk is also linked to the dysbiosis caused by *H. trogontum* (*5,6*). In the present case, we assume that the immunocompetent patient had a chronic colitis due to *H. trogontum* and that she had an episode of acute colitis with bacteremia after several years of intermittent symptoms. The patient is further monitored for IBD, especially because preliminary experiments showed a Caco-2 cell cycle arrest after incubation with the *H. trogontum* isolate and this effect may be involved in the early stage of IBD.

Caco-2/TC7 cells were plated on glass coverslips and grown in Dulbecco modified Eagle medium containing 20% fetal calf serum (DMEM and fetal calf serum; GIBCO, Grand Island, NY, USA) with nonessential amino acids, at 37°C in a CO_2 (10%) incubator. Three days later, cells were challenged with *H. trogontum* overnight, and for 72 hours, in DMEM at 37°C; control cells were incubated in DMEM only or challenged with *H. trogontum* heated for 10 minutes at 100°C. Cells were washed once with phosphate-buffered saline (PBS; GIBCO) and fixed with 3.7% paraformaldehyde. Bacteria were permeabilized with ethanol for 5 min at –20°C, washed three times with PBS, blocked in PBS buffer containing 10% fetal calf serum for 15 min, and incubated with DAPI (4',6-diamidino-2-phenylindole) (1:100) and Alexa Fluor 488 Phalloidin (1:200). Z sections (200 nm) were acquired using spinning-disk confocal microscopy.

Three separate experiments were made with counts of 6,000 cells for each condition. The Caco-2 cell cycle arrest assays yielded similar results with overnight and 72-hour incubations, revealing,

respectively, only 4 or 7 dividing cells/1,000 as opposed to 44 or 45/1,000 cells in the controls ($p<0.001$, by χ^2; Supplemental Figure C, D). This effect, undescribed to date, may be involved during initiation or throughout the early stages of colitis (possibly preceding chronic IBD) as supported by the fact that Caco-2 cell cycle arrest was also observed with dextran sulfate sodium, an inducer of colitis in animal IBD models, and considering that *H. trogontum* is able to initiate IBD in a rodent model (*7*, ref. 3 cited in the article). Repeated intestinal surface damage and injury are considered to be prominent in diverse intestinal disorders, e.g., IBD. The intestinal epithelial cells have a high turnover rate, which could explain why cell cycle arrest may impede barrier functions and absorption (*7*). Extensive studies are necessary to further elucidate the role of NHPH in enteric diseases.

This work has been supported by a grant from the Institut National de la Santé et de la Recherche Médicale.

References

1. Araoka H, Baba M, Kimura M, Abe M, Inagawa H, Yoneyama A. Clinical characteristics of bacteremia caused by *Helicobacter cinaedi* and time required for blood cultures to become positive. J Clin Microbiol. 2014;52:1519–22. PubMed http://dx.doi.org/10.1128/JCM.00265-14

2. Relman DA, Schmidt TM, MacDermott RP, Falkow S. Identification of the uncultured bacillus of Whipple’s disease. N Engl J Med. 1992;327:293–301. PubMed http://dx.doi.org/10.1056/NEJM199207303270501

3. Dewhirst FE, Shen Z, Scimeca MS, Stokes LN, Boumenna T, Chen T, et al. Discordant 16S and 23S rRNA gene phylogenies for the genus *Helicobacter*: implications for phylogenetic inference and systematics. J Bacteriol. 2005;187:6106–18. PubMed http://dx.doi.org/10.1128/JB.187.17.6106-6118.2005

4. Hänninen ML, Utriainen M, Happonen I, Dewhirst FE. Helicobacter sp. flexispira 16S rDNA taxa 1, 4 and 5 and Finnish porcine *Helicobacter* isolates are members of the species *Helicobacter trogontum* (taxon 6). Int J Syst Evol Microbiol. 2003;53:425–33. PubMed http://dx.doi.org/10.1099/ijs.0.02389-0

5. Whary MT, Danon SJ, Feng Y, Ge Z, Sundina N, Ng V, et al. Rapid onset of ulcerative typhlocolitis in B6.129P2–IL10tm1Cgn (IL-10–/–) mice infected with *Helicobacter trogontum* is associated with decreased colonization by altered Schaedler’s flora. Infect Immun. 2006;74:6615–23. PubMed http://dx.doi.org/10.1128/IAI.01091-06

6. Hansen R, Thomson JM, Fox JG, El-Omar EM, Hold GL. Could *Helicobacter* organisms cause inflammatory bowel disease? FEMS Immunol Med Microbiol. 2011;61:1–14. PubMed http://dx.doi.org/10.1111/j.1574-695X.2010.00744.x

7. Araki Y, Sugihara H, Hattori T. In vitro effects of dextran sulfate sodium on a Caco-2 cell line and plausible mechanisms for dextran sulfate sodium-induced colitis. Oncol Rep. 2006;16:1357–62. PubMed

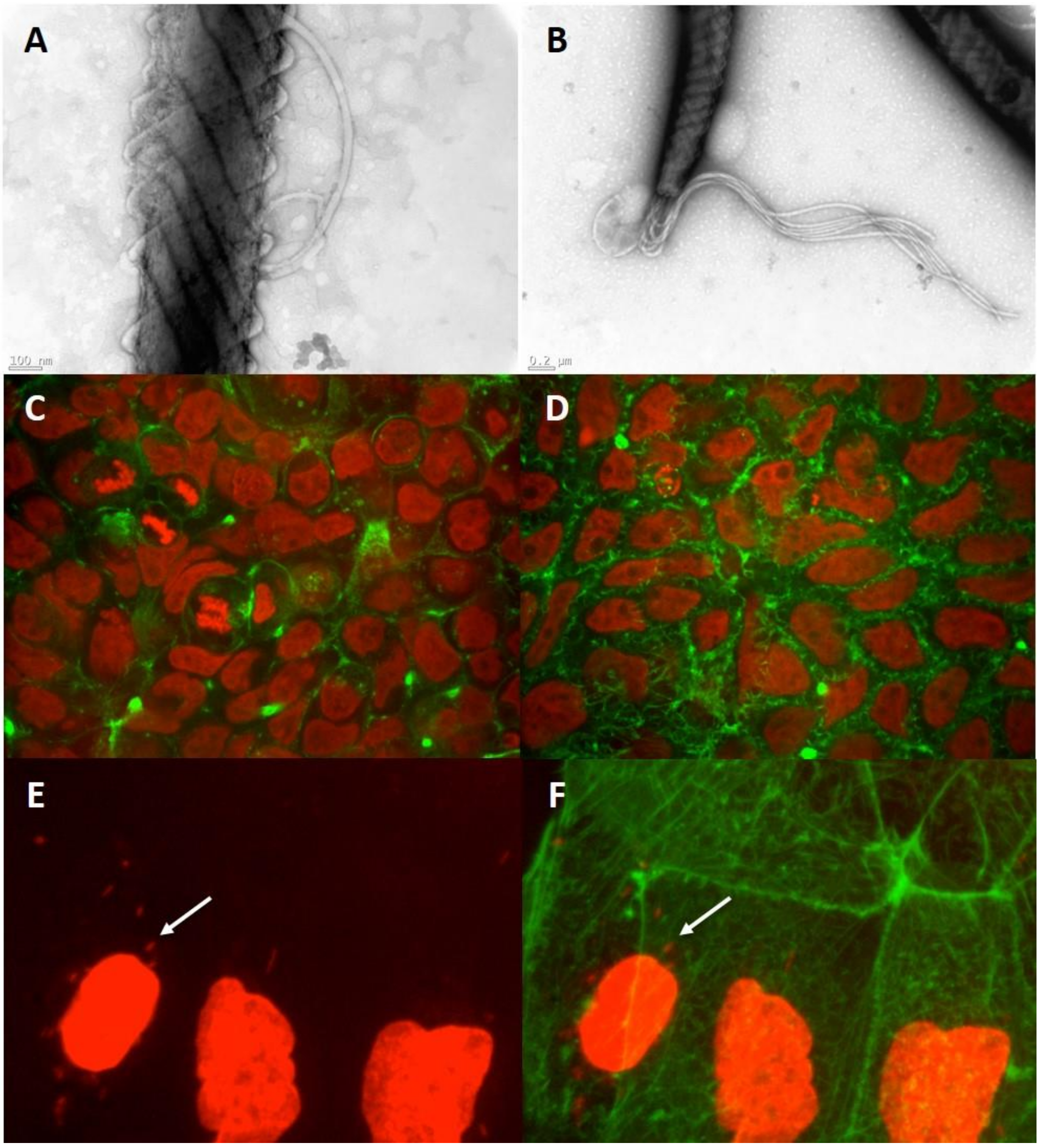

Technical Appendix Figure. Transmission electron micrograph of *Helicobacter trogontum* and Caco-2 cells challenged with *H. trogontum*. Transmission electron micrograph of a negatively stained preparation showing periplasmic fibers coiled around the protoplasmic cylinder (A) and 7 bipolar flagella (B). Representative image of Caco-2 cells with DNA (red, DAPI [4',6-diamidino-2-phenylindole] and actin (green, alexa488-phalloidin) staining. Control cells showing 3 dividing cells (C); Cells challenged with *H. trogontum* showing no division after 12 hours (D). Representative image showing the presence of *H. trogontum* (arrows) in Caco-2 cells. DNA (red, DAPI) staining (E); DNA and actin (green, alexa488-phalloidin) staining (F). Original magnification for panels C–F is ×60.
